# Test Anxiety in Autistic University Students—Preliminary Results from a German-Speaking Sample

**DOI:** 10.3390/brainsci11030390

**Published:** 2021-03-19

**Authors:** Alafia Zavery, Myriam Zäch, Alex Bertrams

**Affiliations:** Educational Psychology Lab, Institute of Educational Science, University of Bern, 3012 Bern, Switzerland; myriam.zaech@edu.unibe.ch (M.Z.); alexander.bertrams@edu.unibe.ch (A.B.)

**Keywords:** autism spectrum, autistic traits, clinical psychology, developmental disorder, educational psychology, psychiatry, test anxiety, university students

## Abstract

Autism is a neurodevelopmental condition associated with increased levels of anxiety. However, whether autism is related to heightened test anxiety as one situation-specific type of anxiety has not yet been examined. This question may be relevant for the achievement and well-being of autistic people in educational settings (e.g., at universities). In this study, we took a first step to investigate whether autistic university students have increased test anxiety. A sample of 16 German-speaking university students completed an established diagnostic instrument to measure test anxiety and its components of worry, emotionality, cognitive interference, and lack of confidence. The scores of the autistic students were compared with the test anxiety means and percentile ranks of the standardization sample for the applied test anxiety measure (*n* = 1350). For an additional comparison, the test anxiety means and percentile ranks of non-autistic university students (*n* = 101) were assessed during the last third of the semester; that is, close to the examinations. Overall, the results suggest that autistic university students have remarkably increased test anxiety. Although the present findings must be considered preliminary, they suggest that text anxiety in educational settings may be a neglected significant problem for autistic people that requires further attention in research and practice.

## 1. Introduction

Autism is a neurodevelopmental condition that is defined by (a) difficulties in the culturally common forms of social interaction and communication and (b) restrictive and repetitive behaviors [1]. Studies have revealed that individuals on the autism spectrum show increased levels of anxiety (e.g., social anxiety [2]), corresponding to the fact that people on the autism spectrum have a higher susceptibility to anxiety at the neuronal level [3]. A well-known specific type of anxiety is test anxiety. Test anxiety research has a long tradition (over 100 years) and has provided various and important insights into educational contexts [4]. Research in the field of autism has been conducted intensively in recent years and could result in diverse findings regarding test anxiety. The link between these two research areas is novel and relevant for the educational–psychological field. At the same time, the educational–psychological field is important and useful for autism research [5]. To date, the relationship between test anxiety and autism has not been explored and remains a research gap.

Generally, anxiety is defined as an aversive emotional experience that is caused by a threat and is characterized by increased arousal, tension, nervousness, and worry [6]. Test anxiety is considered a situation-specific disposition (trait). Persons high in test anxiety tend to react in evaluative exams or other performance situations with increased anxiety [7]. Test anxiety can lead to the avoidance of learning opportunities and learning and performance impairments [4,6,8]. It also reduces students’ well-being [9]. People on the autism spectrum often have diminished well-being [10,11]. For autistic individuals who are in educational contexts, such as university students, test anxiety could exacerbate their reduced well-being and interfere with their educational progress.

Test anxiety is composed of emotional and cognitive components [8,12]. The emotional component includes automatic arousal and the affect caused by the exam situation [12]. The cognitive component includes worries about being evaluated and failing as well as the expectation of aversive consequences [12]. Similar to emotionality, worry is activated in stressful situations (e.g., exams, evaluations [8]). Cognitive interference and lack of confidence are two additional components of test anxiety [7,13] that have been included in the concept by some authors (e.g., [14]). Cognitive interference refers to impaired cognition in test situations, whereas lack of confidence refers to the absence of optimism about one’s performance and self-control during test situations [15]. These four components of test anxiety are included in the present study.

We explored whether adults on the autism spectrum show increased test anxiety compared to non-autistic adults. We assumed that autistic university students have an increased likelihood of being test anxious, as people on the autism spectrum are generally prone to anxiety [2,3], and they are in a phase of life in which test situations are salient and important [4]. Autistic university students are also considered an appropriate examination group because of the limited support services provided to adults on the autism spectrum (especially persons with high-functional autism), and their dropout rate from university might be high [16]. Heightened test anxiety scores in autistic students would point to the need for more specific support for this group.

As a first step in combining autism and test anxiety research, we compared German-speaking autistic university students’ averaged test anxiety scores with the scores of a standardization (norming) sample for an established test anxiety measure. As another comparison sample, we asked non-autistic university students to complete the same test anxiety measure during the last third of the semester; that is, close to the examinations. With the examinations approaching, test anxiety may become more prevalent among test-anxious students and potentially lead to higher self-reports of test anxiety. If autistic students’ test anxiety scores were still higher, this would suggest that they have considerably increased test anxiety.

## 2. Materials and Methods

### 2.1. Participants

The autistic university students were recruited in German-speaking autism internet forums. From a larger sample, the participants who indicated being university students and were formally diagnosed with an autism spectrum condition were selected for the purpose of this study. The sample consisted of 16 university students (56% female, 38% male, and 6% diverse; *M*_age_ = 24.81, *SD*_age_ = 4.52). All of the students indicated having sufficient German language skills.

The autistic university student group was compared with the nonclinical standardization sample of university students for the applied test anxiety measure (*n* = 1350; 59% female and 41% male; *M*_age_ = 22.85, *SD*_age_ = 4.51) [14]. As another comparison group (close-to-examination non-autistic university student sample), 101 university students were recruited at a German university in January 2020 during the last third of the semester (79% female and 21% male; *M*_age_ = 22.47, *SD*_age_ = 3.76). None of these students were diagnosed with an autism spectrum condition, and they completed the same test anxiety measure as the autistic university students in this study.

### 2.2. Procedures

To recruit autistic university students, access to an online survey was published in different German-speaking autism internet forums. For complete access to the autism internet forum, only registered members were allowed. The online survey was programmed on Unipark [17].

The participants were first welcomed and asked for their informed consent. Next, the autistic university students completed the brief version of the Autism-Spectrum Quotient for adults (AQ-10) in German [18,19]. The AQ-10 is a widely applied self-report used as a screening tool for suspected autistic disorder and for measuring the extent of autistic traits in autism research [20,21]. It consists of 10 items capturing autistic core traits (e.g., “I usually concentrate more on the whole picture rather than the small details” and “I find it easy to do more than one thing at once”). Each item was rated on a four-point Likert-type scale. Definitely/slightly agree and definitely/slightly disagree are awarded one and zero points, respectively (or zero/one point when the items are reversed), which are summed to form the total score. Higher scores reflect more pronounced autistic traits.

Following the AQ-10, the autistic university students completed the standardized Prüfungsangstfragebogen (PAF, test anxiety questionnaire) [14], which is well validated, designed for university students, and frequently used in German-speaking areas. The PAF contains 20 items in total. The four subdimensions of test anxiety, namely, worry (e.g., “I think about how important the exam is to me”), emotionality (e.g., “I feel anxious”), cognitive interference (e.g., “Suddenly, thoughts shoot through my head that block me”), and lack of confidence (e.g., “I trust in my performance,” reversed), are measured with five items each. Each item was rated on a four-point Likert-type scale (1 = almost never to 4 = almost always). Higher scores represent higher test anxiety. Afterwards, sociodemographic information was requested, and the autistic participants were thanked and carefully debriefed.

The close-to-examination non-autistic university student sample was recruited by approaching students who happened to be on the campus of a German university at the time and asking them to participate in a short questionnaire study. After giving their informed consent, the students provided sociodemographic information and completed the paper-and-pencil version of the PAF. Afterwards, they were thanked and debriefed.

In this study, Cronbach’s α for the AQ-10 was satisfactory (0.74) in the autistic university student sample. The Cronbach’s αs for the total test anxiety score across the 20 PAF items were high for the autistic university student sample (0.93), the PAF standardization sample (0.88), and the close-to-examination non-autistic university student sample (0.89). The Cronbach’s αs for the PAF subscales were also high for the autistic university student sample, the PAF standardization sample, and the close-to-examination non-autistic university student sample (worry: 0.93/0.81/0.85; emotionality: 0.92/0.86/0.87; cognitive interference: 0.91/0.79/0.78; lack of confidence: 0.88/0.85/0.86).

### 2.3. Analysis Strategy

The following *t*-tests refer to the specific means in the sample of autistic university students and the corresponding means in the two non-clinical comparison samples (Table 1). Bootstrapping was applied to test the robustness of the mean differences. Bootstrapping is a technique for taking repeated samples (with replacements) from the present dataset, which allows calculation of confidence intervals for the statistic of interest (in the present case, the difference between two means) [22]. Whenever bootstrapping was used in this study, it was bias-corrected and accelerated (BCa), and it was based on 1000 bootstrap samples [22]. The percentile ranks according to the test anxiety questionnaire manual [14] for the different samples were also reported for interpretation purposes. According to the manual, percentile ranks between 25 and 75 are considered unremarkable, whereas percentile ranks less than 25 and more than 75 are considered remarkably low and high, respectively [14].

## 3. Results

### 3.1. Total Test Anxiety Scores

Test anxiety was significantly higher in the autistic university student sample than in the standardization sample (mean difference = 0.67, BCa 95% CI [0.31, 0.98], *t*[15] = 3.93, *p* = 0.001, *d* = 0.98) and the 2020 close-to-examination sample (mean difference = 0.46, BCa 95% CI [0.10, 0.77], *t*[15] = 2.69, *p* = 0.02, *d* = 0.67). The percentile ranks were 91 for the autistic student sample, 56 for the standardization sample, and 71 for the 2020 close-to-examination sample.

### 3.2. Worry Subscale Scores

The worry scores of the autistic university student sample did not differ from the worry scores of the standardization sample (mean difference = 0.32, BCa 95% CI [−0.17, 0.74], *t*[15] = 1.24, *p* = 0.23, *d* = 0.31) and the 2020 close-to-examination sample (mean difference = 0.05, BCa 95% CI [−0.46, 0.51], *t*[15] = 0.18, *p* = 0.86, *d* = 0.04). The percentile ranks were 72 for the autistic student sample, 51 for the standardization sample, and 61 for the 2020 close-to-examination sample.

### 3.3. Emotionality Subscale Scores

Autistic university students had higher emotionality scores than the university students in the standardization sample (mean difference = 0.83, BCa 95% CI [0.36, 1.23], *t*[15] = 3.73, *p* = 0.002, *d* = 0.93) and the 2020 close-to-examination sample (mean difference = 0.65, BCa 95% CI [0.24, 1.05], *t*[15] = 2.92, *p* = 0.01, *d* = 0.73). The percentile ranks were 86 for the autistic student sample, 56 for the standardization sample, and 66 for the 2020 close-to-examination sample.

### 3.4. Cognitive Interference Subscale Scores

Autistic university students were more likely to experience cognitive interference than the university students in the standardization sample (mean difference = 0.85, BCa 95% CI [0.28, 1.38], *t*[15] = 3.60, *p* = 0.003, *d* = 0.90) and the 2020 close-to-examination sample (mean difference = 0.58, BCa 95% CI [0.15, 0.95], *t*[15] = 2.45, *p* = 0.03, *d* = 0.61). The percentile ranks were 89 for the autistic student sample, 57 for the standardization sample, and 68 for the 2020 close-to-examination sample.

### 3.5. Lack of Confidence Subscale Scores

Autistic university students had higher lack of confidence scores than the university students in the standardization sample (mean difference = 0.69, BCa 95% CI [0.30, 1.04], *t*[15] = 3.56, *p* = 0.003, *d* = 0.89) and the 2020 close-to-examination sample (mean difference = 0.58, BCa 95% CI [0.21, 0.90], *t*[15] = 2.99, *p* = 0.009, *d* = 0.75). The percentile ranks were 86 for the autistic student sample, 56 for the standardization sample, and 56 for the 2020 close-to-examination sample.

### 3.6. Autistic Traits

The extent of autistic university students’ autistic traits correlated strongly with the total test anxiety scores (*r*[14] = 0.80, *p* < 0.001), the worry subscale scores (*r*[14] = 0.63, *p* = 0.008), the emotionality subscale scores (*r*[14] = 0.77, *p* < 0.001), and the cognitive interference subscale scores (*r*[14] = 0.61, *p* = 0.01) but not with the lack of confidence subscale scores (*r*[14] = 0.32, *p* = 0.22).

## 4. Discussion

The aim of this study was to examine whether test anxiety is increased in German-speaking autistic university students. Overall, autistic participants had a higher total test anxiety score, a higher emotionality subscale score, a higher cognitive interference subscale score, and a higher lack of confidence subscale score compared with the standardization sample in an established test anxiety diagnostic questionnaire for German-speaking university students [14]. These scores were also higher for the autistic participants compared with a sample of university students who reported their test anxiety during the last third of the semester (i.e., close to the examinations). Total test anxiety, emotionality, cognitive interference, and lack of confidence were remarkably high in the autistic participants, as the sample’s percentile ranks exceeded the 75% threshold [14], indicating a problematically strong text anxiety, on average. In the comparison samples, the percentile ranks were in the unremarkable range, even for those individuals who were close to their examinations.

Interestingly, autistic participants’ worry subscale scores were unremarkable. Thus, the tendency to worry in test situations was not the cause of their relatively high total test anxiety. This null result suggests that the reasons for increased anxiety in autistic people, even beyond the test anxiety domain [23], are not primarily found at the cognitive level. For the other facets of test anxiety, explanations for the higher scores in autistic people, apart from worrying, are available. High test-anxious emotionality may be explained by neuronally caused hyper-excitability in autistic people [3]. An increased tendency for cognitive interference may stem from autistic people’s difficulties in executive functioning, including a decreased ability for selective attention [24]. Regarding lack of confidence, autistic people have recently been shown to be less self-deceptive than non-autistic people [25], which may result in their lower self-confidence [26].

We also found that autistic university students’ extent of autistic traits was strongly associated with higher total test anxiety and three of the test anxiety subscale scores. Thus, even within the autistic population, the severity of autistic symptoms may be relevant for the degree of test anxiety. However, we do not want to overstate these relationships, as very recent research has revealed some psychometric deficits of the 10-item AQ that we used in this study [27,28].

With respect to the implications, the present findings suggest that test anxiety can be added to the specific anxieties that have been shown to be increased in autistic people, such as social anxiety [2]. The extensive test anxiety literature [4] may be relevant and useful for autism researchers to understand autistic individuals’ experiences in the educational domain. The test anxiety literature also provides well-examined interventions for test-anxious people, such as anxiety management training or cognitive-attentional training [4]; potentially, these interventions may be useful for autistic students. Universities that offer counselling for autistic students could consider their increased test anxiety and offer possible solutions.

The present results must be considered preliminary because of the limitations of the study, which threaten internal, external, and conclusion validity. The sample of autistic university students was small and limited to German-speaking university students. Test anxiety was assessed by self-reports, which usually may be biased by socially desirable response tendencies. Autistic people seem to have a lowered tendency to self-deceive [25], which could influence their self-reports of test anxiety. In addition, although we used an established diagnostic instrument, we measured test anxiety at a general level only. It has been shown that test anxiety for oral and written exams can be differentiated [29]. Possibly, autistic university students are more anxious during oral examinations, as their difficulties in communication and social interaction may be a significant additional burden during the test situation.

Future research may seek to overcome these limitations. The self-regulation of test anxiety [30,31] may also be a relevant topic for further research, considering that autistic people often have difficulties in executive functioning [32,33]. In this regard, research should also examine which of the previously developed and scientifically evaluated interventions for test anxious people [4] are most useful for autistic university students. Given their increased levels of the test anxiety components emotionality and cognitive interference, autistic university students might benefit from emotion-focused regulation techniques (e.g., anxiety management training) as well as cognitive-focused interventions improving attentional control (e.g., cognitive-attentional training). Moreover, the differences between male and female autistic students require further investigation. Finally, whether the present findings can be replicated, for instance, in samples of school students, is of interest.

## Figures and Tables

**Table 1:** Means and Standard Deviations (in brackets) of Test Anxiety Scores in the Autistic University Student Sample, the PAF Standardization Sample [14], and the Close-to-Examination Non-autistic University Student Sample (January 2020).

	Autistic University Students	PAF Standardization Sample	2020 University Student Sample
	(*n* = 16)	(*n* = 1350)	(*n* = 101)
Total test anxiety score	2.86 (0.68)	2.19 (0.46)	2.40 (0.50)
Worry subscale	2.93 (1.02)	2.61 (0.67)	2.88 (0.73)
Emotionality subscale	2.85 (0.89)	2.02 (0.72)	2.20 (0.76)
Cognitive interference subscale	2.63 (0.94)	1.78 (0.60)	2.05 (0.66)
Lack of confidence subscale	3.03 (0.77)	2.34 (0.62)	2.45 (0.61)

Note. PAF = Prüfungsangstfragebogen (test anxiety questionnaire). Scores were obtained by averaging the item responses in a (sub)scale. The responses were given on the following scale: 1 (almost never), 2 (sometimes), 3 (often), and 4 (almost always).

## Data Availability

The data supporting the conclusions of this article will be made available by the authors.
